# Circulating miRNAs as Diagnostic and Prognostic Biomarkers in High-Grade Gliomas

**DOI:** 10.3389/fonc.2022.898537

**Published:** 2022-05-12

**Authors:** Jianing Wu, Abdulrahman Al-Zahrani, Ozal Beylerli, Rinat Sufianov, Rustam Talybov, Svetlana Meshcheryakova, Galina Sufianova, Ilgiz Gareev, Albert Sufianov

**Affiliations:** ^1^ Department of Neurosurgery, Shenzhen University General Hospital, Guangdong, China; ^2^ Department of Neurosurgery, Sechenov First Moscow State Medical University (Sechenov University), Moscow, Russia; ^3^ Department of Neurosurgery, King Saud Medical City (KSMC), Riyadh, Saudi Arabia; ^4^ Department of Neurosurgery, Federal Center of Neurosurgery, Tyumen, Russia; ^5^ Department of Radiology, Federal Center of Neurosurgery, Tyumen, Russia; ^6^ Department of General Chemistry, Bashkir State Medical University, Ufa, Russia; ^7^ Department of Pharmacology, Tyumen State Medical University, Tyumen, Russia

**Keywords:** high-grade glioma, circulating, diagnosis, prognosis, biomarker, miRNA

## Abstract

**Objectives:**

miR-181a/b and miR-410 downregulation and miR-155 upregulation has been shown to play important roles in the oncogenesis and progression of gliomas including high-grade gliomas. However, the potential role of plasma miR-181a/b, miR-410 and miR-155 in the diagnosis and prognosis of high-grade gliomas remains poorly known.

**Methods:**

We retrieved published articles from the PubMed, the Cochrane Central Register of Controlled Trials, and Web of Science database and obtained different sets of data on microRNAs (miRNAs) expression profiling in glioma and highlighted the most frequently dysregulated miRNAs and their gene-targets (PDCD4, WNT5A, MET, and EGFR) in high-grade gliomas. Quantitative reverse transcription polymerase chain reaction (qRT-PCR) was carried out to measure the pre- and postoperative plasma levels of miR-181a/b, miR-410 and miR-155 in 114 Grade 3-4 glioma patients, 77 Grade 1-2 glioma patients and 85 healthy volunteers as control group. The diagnostic and prognostic value of circulating miR-181a/b, miR-410 and miR-155 as biomarker was estimated by the Receiver Operating Characteristic (ROC) curve and the area under the curve (AUC) and Kaplan–Meier analysis.

**Results:**

We found a plasma miRNA signature including three downexpressed miRNAs and one overexpressed (miR-181a, miR-181b and miR-410; miR-155) in high-grade glioma patients in comparison with low-grade glioma patients control group. The ROC curve AUC of these four circulating miRNAs were ≥ 0.75 for high-grade glioma patients in before and after surgery. Higher circulating miR-155 and lower miR-181a/b and miR-410 expression is associated with clinical data, clinic pathological variables, worse overall survival (OS) of patients and negative correlated with potential gene-targets expression. Moreover, Kaplan–Meier analysis showed that miR-181a/b, miR-410 and miR-155 were independent predictors of OS in high-grade glioma patients.

**Conclusions:**

Our data, for the first time, demonstrated that circulating miR-181a/b, miR-410 and miR-155 could be a useful diagnostic and prognostic non-invasive biomarkers in high-grade gliomas.

## 1 Introduction

In recent decades, there has been a steady trend towards an increase in both the general oncological incidence in general and the general incidence of central nervous system (CNS tumors) tumors in particular. Gliomas are the most common and aggressive type of primary tumor in adults. The largest proportion among CNS tumors are gliomas (50–55%), which are malignant in 50–70% of cases (1). According to the 2021 World Health Organization (WHO) classification of CNS tumors, gliomas were classified into four main histological groups (Grades 1-4) according to their microscopic characteristics (such as cytological atypia, anaplasia, mitotic activity, microvascular proliferation and necrosis) and clinical manifestations (2). Glioblastoma (WHO Grade 4) and anaplastic astrocytoma (WHO Grade 3), a type of high-grade gliomas, are the most common primary brain tumors, affecting patients of all ages. Despite all the achievements of modern medicine, the prognosis for patients with high-grade gliomas remains unsatisfactory. So, with anaplastic astrocytomas, the average life expectancy is 2-3 years, and with glioblastomas - from 8 to 15 months (3). In this regard, in order to improve the diagnosis and optimization of ongoing therapy, as well as predict the course of the disease and understand the mechanisms of oncogenesis, an active search is being carried out for biomarkers that are informative and public accessibility. The possibility of accurate diagnosis and prognosis of the course of the disease, along with the improvement of the treatment of patients with high-grade gliomas is an urgent problem. Despite significant recent advances in the diagnosis of gliomas using various modifications of imaging techniques followed by histopathological examination, tumor detection is still limited by its size and location, as well as by the heterogeneity of its tissue (4). In this regard, it is necessary to develop new diagnostic approaches that, together with the available methods, will improve the accuracy of diagnosis. A promising approach for diagnosis in CNS tumors is fluid biopsy, which involves finding and measuring the level of various circulating biomolecules in human body fluids, such as blood or cerebrospinal fluid (CSF).

MicroRNAs (miRNAs) are small non-coding RNAs consisting of 18-20 nucleotides that play an important role in the regulation of gene expression at the post-transcriptional level by interacting with 3’-untranslated regions (3’-UTR) of messenger RNAs (mRNA)-targets (5). MiRNAs are involved in the regulation of such physiological processes as cell proliferation, differentiation, apoptosis, angiogenesis, etc. MiRNAs play an important role in the regulation of both physiological processes (5). On the other hand, miRNAs have been found to be involved in the oncogenesis of many human tumors, acting as tumor suppressors or oncogenes (6). MiRNAs are capable of influencing all events considered in terms of tumor progression, including tumor growth, invasion, metastasis, and angiogenesis. The significance of miRNAs in high-grade gliomas has been proven both by changes in their expression and by dysregulation of the expression of mRNA-targets (7). MiR-155is one of the most well-known oncogenic miRNA, miR-155 overexpression has been documented in gliomas, extraordinarily in high-grade glioma cells and tissue. Wu et al. in their research found that the high level of miR-155 in U87-MG cell line can promote the proliferation, invasion and migration of tumor cells, inhibit their senescence and apoptosis, and activate the phosphatidylinositol 3-kinase (PI3K)/AKT signaling pathway (8). In other study results showed that the level of miR-155 was up-regulated in glioma patients, accompanied by high pathological grade (9). In addition, miR-155 may activates the growth of U87 glioma cells and increases the sensitivity of glioblastoma to temozolomide (TMZ) by targeting Six1 *in vivo* (10).

Some number of studies about miR-181a/b and miR-410 therapeutics have been carried out, and verified its tumor-suppressive role in gliomas. For instance, the expression of miR-181 family members has been decreased in glioma (11). While miR-181c has been the most down-regulated one in the WHO Grade I gliomas, miR-181a/b exhibited the fastest decrease rate, with a significant decrease in the glioblastoma (11, 12). Forced up-regulation of miR-181a/b remarkably suppressed high-grade glioma cell lines (U87, TJ905, and U251) tumor growth, proliferation, invasion and promote tumor cells apoptosis (13). In the other research, Chen et al. demonstrated that high-grade glioma expressed comparatively higher MET expression and lower miR-410 while low-grade glioma with lower MET and higher miR-410 (14). The authors concluded that miR-410 directly targets 3’-UTR of MET mRNA and may affect high-grade glioma cell proliferation and invasion through MET regulated AKT signaling.

It is known that circulating miRNAs are in a stable form and are detected in human biological fluids such as blood, urine, cerebrospinal fluid (CSF) and saliva. In this regard, circulating miRNAs are considered as new biomarkers of interest in a number of diseases, including tumors (15). Circulating miR-181a/b, miR-410 and miR-155 can be released into the biological fluids in response to activation of process oncogenesis. Thus, the present study examined the expression profile of these circulating miRNAs in the plasma of patients with high-grade glioma. Furthermore, we evaluated the association among gene-targets expression, clinical data, clinic pathological variables, and diagnostic or prognosis value.

## 2 Materials and Methods

### 2.1 Patients and Clinical Samples

**Figure 1:**
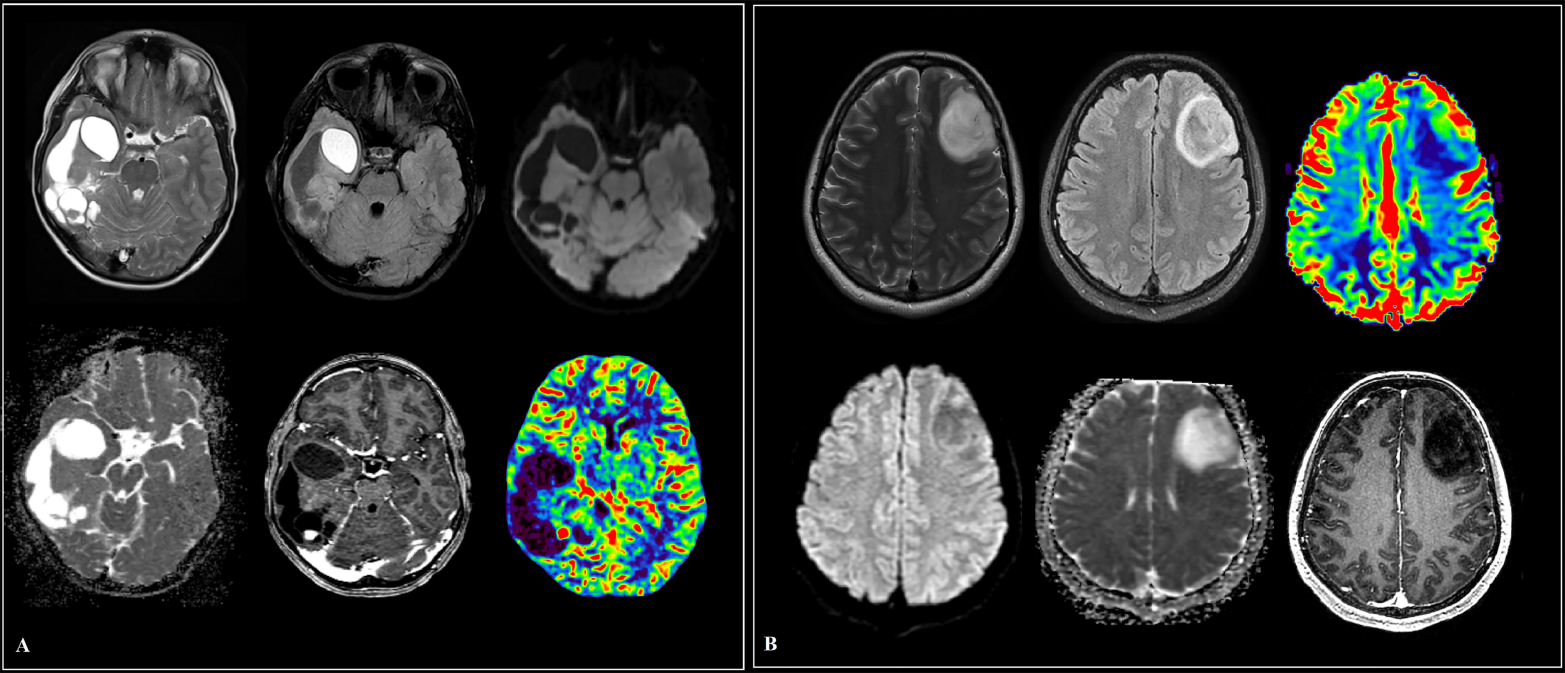
Cases of low-grade glioma patients **(A, B)**. **(A)** A large intracerebral tumor of the right temporo-occipital areas with a heterogeneous cystic-solid structure, contrasted as a group of nodules and small closed rings in the solid part. The contents of the cysts are similar to the cerebrospinal fluid in terms of signal characteristics, with the exception of a cyst in the pole of the temporal lobe, where a protein admixture and traces of hemorrhage are detected, the walls of the cysts do not increase. Diffusion restriction in the structure of education is not determined. In the posterior sections, a node with elevated median cerebral blood volume (CBV) values according to multi-slice computed tomography (MSCT) perfusion. **(B)** Predominantly in the middle frontal gyrus of the left frontal lobe, an intracerebral mass with diffuse distribution, clear contours and a sign of T2/fluid attenuation inversion recovery (FLAIR) mismatch. The tumor involves white and gray matter with “swelling” of the cortical plate. There is no restriction of diffusion, contrasting in the formation. Reliable areas of hyperperfusion according to dynamic susceptibility contrast (DSC) MR perfusion are not determined.

**Figure 2:**
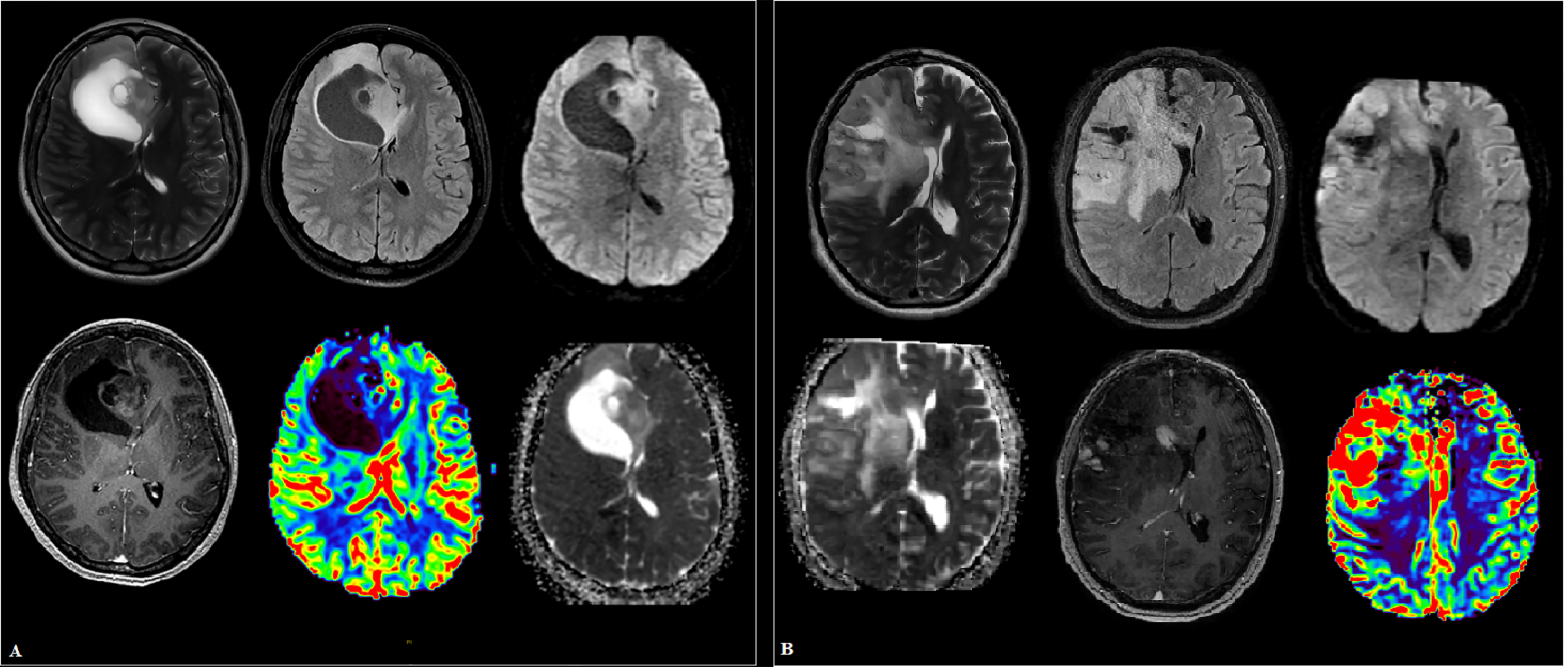
Cases of high-grade glioma patients **(A, B)**. **(A)** Intracranial tumor of the right frontal lobe and genu corpus callosum, in addition to the white matter, involving the cortical plate with a mass effect on the anterior horn of the right lateral ventricle. The parasagittal solid component has small foci of hyperperfusion on dynamic susceptibility contrast (DSC) MR perfusion and areas of increased cellularity in apparent diffusion coefficient (ADC), is heterogeneous in signal in T2 weighted image (T2WI), and shows heterogeneous contrast enhancement. **(B)** A large “intra-axial” area of in homogeneously elevated signal in T2 and fluid attenuation inversion recovery (FLAIR) in the right frontal lobe and basal ganglia, extending into the gray matter, the opposite hemisphere along the genu corpus callosum, with a group of subpial (and one subependial) nodules of homogeneous intense contrast enhancement. Against the background of uneven diffusion, zones of increased relative cerebral blood volume (rCBV) are revealed along the lateral contour of the right frontal lobe.

We enrolled 301 subjects in this study from January 2019 to August 2020 in Federal Center of Neurosurgery (Tyumen, Russia), including 85 healthy volunteers as control group and 191 newly diagnosed glioma patients with various stages. Glioma patients were diagnosed by brain magnetic resonance imaging (MRI), computed tomography (CT) perfusion imaging (Canon Aquilion One, Iomeron 400 mg – 50 ml), dynamic susceptibility contrast (DSC) perfusion (General Electric Discovery W750 3T, Gadovist 7.5 ml) (**Figures 1A, B**, **2A, B**) and histological examination based on the WHO categories, and all patients were classified according to 2021 WHO classification system, including 16 cases of pilocytic astrocytoma (Grade 1), 61 diffuse astrocytoma (Grade 2), 76 cases of anaplastic astrocytoma (Grade 3), and 38 cases of glioblastoma (Grade 3) (2). All patients were grouped into low-grade (WHO Grade 1–2, 77/191) or high-grade (Grade 3–4, 114/191). Plasma samples of patients with low-grade gliomas were collected 3 days before medical preparation for the surgical intervention. Plasma samples of patients with high-grade gliomas were collected twice: 1) 3 days before medical preparation for the surgical intervention; and 2) 10 days after the surgery, usually on the day of discharge from the hospital. None of the patients had received chemotherapy or radiotherapy prior to surgery. In addition, we excluded patients with other tumors, cardiovascular diseases, immune diseases, injuries, organ failure, and infections in their past medical history since these diseases may influence the levels of circulating miRNAs in our patients. All patients with high-grade were followed up at intervals of 1 month in the initial 1–2 years and every 3 months thereafter. Clinical follow-up of 114 patients was finished by August 2021 (44 months). Overall survival time was defined as the period between the initial operation and death, and disease-free survival was the period between the initial operation and tumor recurrence or death. This study was approved by the Ethics Committee of Federal Center of Neurosurgery (Tyumen, Russia) and implemented in accordance with the principles of the Helsinki Declaration. Written informed consent was obtained from all subjects. Patient characteristics are summarized in **Table 1**.

**Table 1:** Clinicopathological characteristics of glioma patients for the study of circulating miRNAs.

Clinical variables (n = 191)	No. of cases	P value
Age		0.002
<50	83	
≥50	108	
Sex		0.09
Male	141	
Female	50	
Tumor size (cm)		0.013
≤ 3	132	
> 3	59	
WHO grade		<0.001
Low-grade (I-II)	77	
High-grade (III-IV)	114	
Extent of resection for high-grade gliomas patients		<0.05
Total	71	
Partial	43	
Tumor location		0.40
Supratentorial	171	
Infratentorial	20	
KPS score		0.008
<90	68	
≥90	123	
Recurrence for high-grade gliomas patients		0.039
Yes	77	
No	37	

WHO, World Health Organization; KPS score, Karnofsky Performance Scale.

### 2.2 Study Design

**Figure 3:**
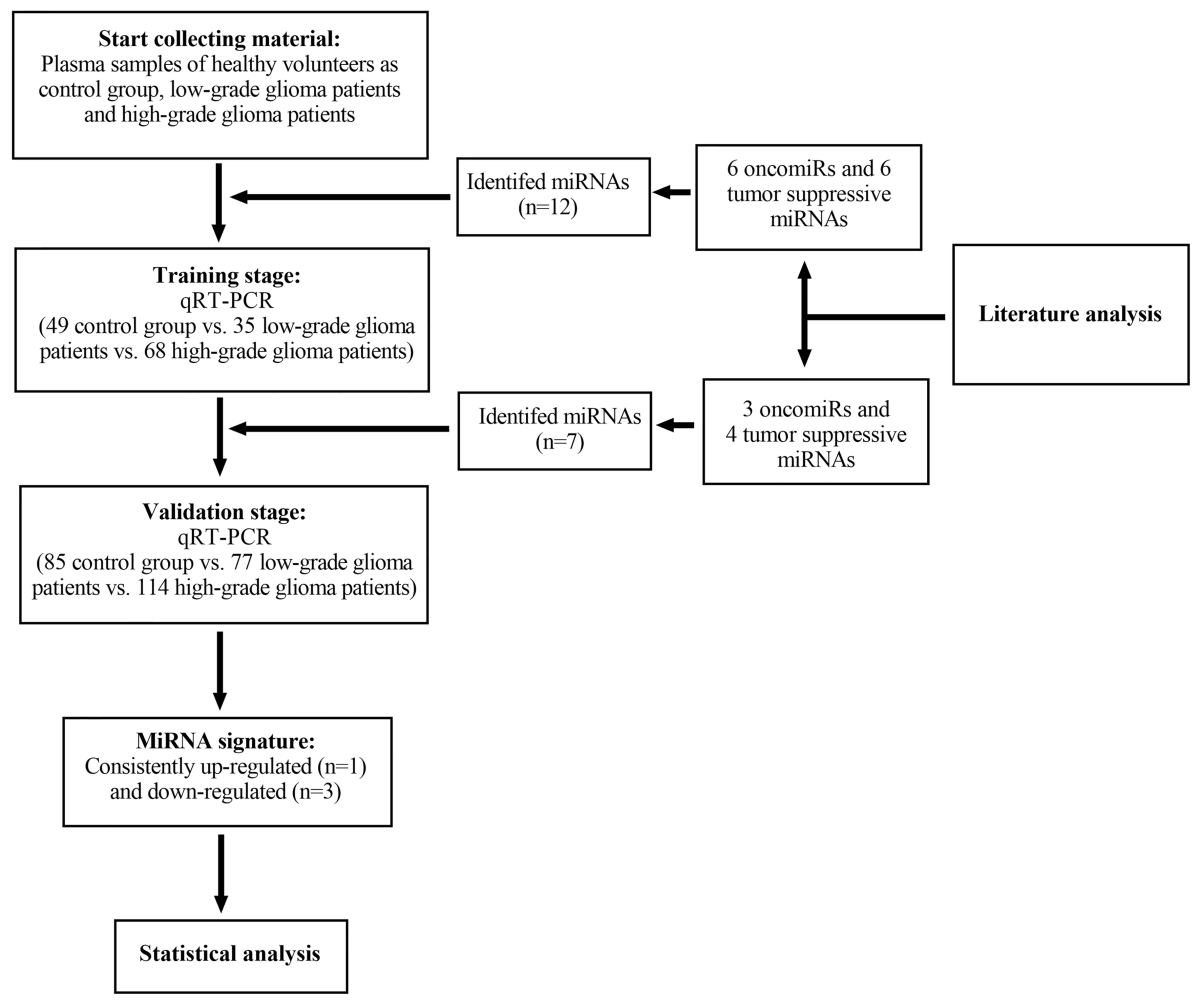
Flow diagram illustrating the steps for microRNAs (miRNAs) selection, expression profiling and differential expression analysis. qRT-PCR, quantitative reverse transcription polymerase chain reaction; miRNAs, microRNAs.

The study was separated into four steps. We performed a comprehensive search for original articles demonstrating the dysregulated miRNAs and their gene-targets in high-grade gliomas. Databases including PubMed, the Cochrane Central Register of Controlled Trials, and Web of Science were used to obtain all relevant studies up to February 2021. Keywords including “glioma” or “high-grade glioma” or “anaplastic astrocytoma” or “glioblastoma” or “malignant brain tumors” or “primary”, “microRNA” or “miRNA” or “miR” or “non-coding RNAs” or “cell-free” or “circulating” or “diagnosis” or “prognosis” or “biomarker” and “gene-targets” or “epigenetic regulation” or “oncogenesis” were used. However, our current study did not find statistically significant identified 4 from 12 circulating miRNAs expression in plasma samples of glioma patients and control group. The flow diagram of the study design is shown in **Figure 3**.

### 2.3 Plasma Preparation

All 10 ml venous blood was collected into tubes containing ethylenediaminetetraacetic acid (EDTA) and centrifuged, where hemolyzed blood samples were excluded. After the first centrifugation at 1600 x g for 10 min at 4°C, the supernatants were carefully removed and transferred to a new tube follow by centrifugation again at 16,000 x g for 10 min at 4°C to remove residual blood cells. Plasma was then stored at −80°C until further processing.

### 2.4 Total RNA Extraction

Total RNA was extracted from 200 μL plasma samples of all glioma patients and healthy controls using the miRNeasy Serum/Plasma Kit for purification of total RNA, including miRNA (Qiagen, Germany) and QIAzol Lysis Reagent (Qiagen, Germany) according to the manufacturer’s instructions. Total RNA purity and concentration were determined using a NanoDrop 2000 spectrophotometer (Thermo Scientific, USA) and consistently yielded A260:A280 and A260:A230 ratios close to 2.0. All isolated total RNA was stored at a −80°C freezer until use.

### 2.5 Synthesis of Complementary DNA

cDNA was synthesized using Transcriptor First Strand cDNA Synthesis Kit (Roche, Germany) by reverse transcription according to the manufacturer’s instructions. Reverse transcription was carried out in 20 μl solution that contained 0.5 μl 20 U/μl Transcriptor reverse transcriptase, 4μl Transcriptor RT Reaction Buffer (5x concentrated), 2 μl Deoxynucleotide Mix, 0.5 μl 40 U/μl protector RNase inhibitor, 2 μl 10 mmol/ml stem-loop RT primers (Invitrogen), 7 μl DEPC water (Invitrogen, USA), and 4 μl total RNA template. After being mixed gently, the reaction mixtures were incubated at 25°C for 10 min, 55°C for 30 min and then 85°C for 5 min. The final cDNA products were stored at −20°C until use. U6 and glyceraldehyde-3-phosphate dehydrogenase (GAPDH) were used as the reference genes. The sequence of all primers used in the present study is provided in **Table 2**.

**Table 2:** Sequence of all primers.

miRNA/Gene-target/Reference gene	Primer Sequence (5’-3')
miR-181a	RT:GTCGTATCCAGTGCAGGGTCCGAGGTATTCGCACTGGATACGACACTCACCGA Forward: GCCCGAACATTCAACGCTGT Reverse: GTGCAGGGTCCGAGGT
miR-181b	RT:CCTGTTGTCTCCAGCCACAAAAGAGCACAATATTTCAGGAGACAACAGGACCCACC; Forward: CGCCGAACATTCATTCATTGCTGTC Reverse: CAGCCACAAAAGAGCACAAT
miR-410	RT:GTCGTATCCAGTGCAGGGTCCGAGGTATTCGCACTGGATACGACACAGGCCA Forward: GTCAGCGCAATATAACACAG Reverse: GTGCAGGGTCCGAGGT
miR-155	RT:CCTGTTGTCTCCAGCCACAAAAGAGCACAATATTTCAGGAGACAACAGGACCCCTA Forward: CGCCGTTAATGCTAATCGTGA Reverse: CAGCCACAAAAGAGCACAAT
PDCD4	Forward: GAAGGTTGCTGGATAGGC Reverse: ATAAACACAGTTCTCCTGGTCATCA
WNT5A	Forward: GTGCAATGTCTTCCAAGTTCTTC
MET	Reverse: GGCACAGTTTCTTCTGTCCTTG
EGFR	Forward: AACACCCTGGTCTGGAAGTACG Reverse: TCGTTGGACAGCCTTCAAGACC
GAPDH	Forward: GTCTCCTCTGACTTCAACAGCG Reverse: ACCACCCTGTTGCTGTAGCCAA
U6	Forward: CTCGCTTCGGCAGCACA Reverse: AACGCTTCACGAATTTGCGT

miRNA, microRNA; RT, reverse transcription; miR, microRNA; WNT5A, Wnt family member 5A; PDCD4, programmed cell death 4; DFFA, DNA fragmentation factor subunit alpha; EGFR, epidermal growth factor receptor; GAPDH, GAPDH, glyceraldehyde-3-phosphate dehydrogenase.

### 2.6 Quantitative Reverse Transcription Polymerase Chain Reaction

qRT-PCR was carried out following the manufacturer’s protocol of FastStart Universal SYBR Green Master (Rox) (Roche, Germany) with 2 μL cDNA template. The PCR mixture (18 μL) contains 10 μL SYBR Green (Rox) (Roche, Germany), 1 μL 10 mmol/mL forward primer (Invitrogen, USA), 1 μL 10 mmol/mL reverse primer (Invitrogen, USA) and 6 μL DEPC water (Invitrogen, USA). PCR reaction was performed in duplicates. All PCR reactions were carried out on an ABI 7500 Real-Time PCR machine (Thermo Fishers, USA). Reaction conditions were 95°C for 10 minutes, followed by 40 cycles of 95°C for 10 s and 60°C for 10 seconds. U6 and GAPDH were used as the reference genes. The sequence of all primers used in the present study is provided in **Table 2**.

### 2.7 Statistical Analysis

Relative levels of the circulating miR-181a/b, miR-410 and miR-155 were quantified using the 2-ΔΔCq method. ROC curves and the area under the curve (AUC) was applied to analysis the diagnostic values of the circulating miR-181a/b, miR-410 and miR-155. Kaplan–Meier analysis was used to generate and analyze survival time data. The univariate Cox proportional hazards regression was used for univariate and multivariate analyses. The Student t-test, ANOVA, chi-square analysis, or Mann-Whitney test was applied, where appropriate. A probability of p<0.05 (*) or p<0.001 (**) or p<0.0001 (***) was considered statistically significant. The statistical analyses were carried out with the IBM SPSS 13.0 software and the graphs were generated by using Graphpad Prism 7.0.

## 3 Results

### 3.1 Detection of Circulating miRNAs in Plasma Samples From Glioma Patients Before Surgery

**Figure 4:**
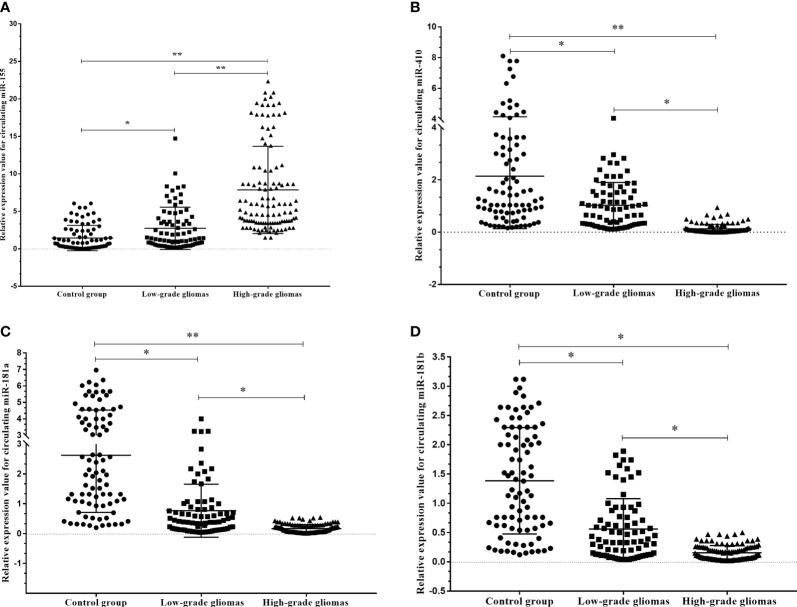
Circulating miR-181a/b, miR-410 and miR-155 expression levels within different grade gliomas and control group **(A–D)**. **(A)** The expression levels of circulating miR-155 in the plasma of high-grade glioma patients were significantly higher than in low-grade glioma patients and control group. In the same time, **(B–D)** the expression levels of circulating miR-181a, miR-181b and miR-410 in the plasma of high-grade glioma patients were significantly lower than in low-grade glioma patients and control group. A probability of p < 0.05 (*) or p < 0.001 (**) was considered statistically significant.

To verify the expression profiles of circulating miR-181a/b, miR-410 and miR-155 in presurgery plasma samples of glioma patients, we detected the expression levels of circulating miR-181a/b, miR-410 and miR-155 in 114 high-grade glioma patients with compare 77 low-grade glioma patients and 85 subjects as control group using qRT-PCR. The results of qRT-PCR assay in **Figures 4A–D** show that the expression levels of circulating miR-155 in plasma of high-grade glioma patients was significantly higher than in low-grade glioma patients and control group (p < 0.05, p<0.001). However, the expression levels of circulating miR-181a/b and miR-410 in plasma of high-grade glioma patients was significantly lower than in low-grade glioma patients and control group (p < 0.05, p<0.001). Our results indicated that increased circulating miR-155 and decreased miR-181a/b and miR-410 levels might play a role in glioma oncogenesis particularly high-grade gliomas. In this case, miR-155 may acts as a potential oncomiR were miR-181a/b and miR-410 may play as a potential tumor suppressive role.

### 3.2 Circulating miRNAs Expression in Before and After Surgery Plasma Samples From Patients With High-Grade Glioma

**Figure 5:**
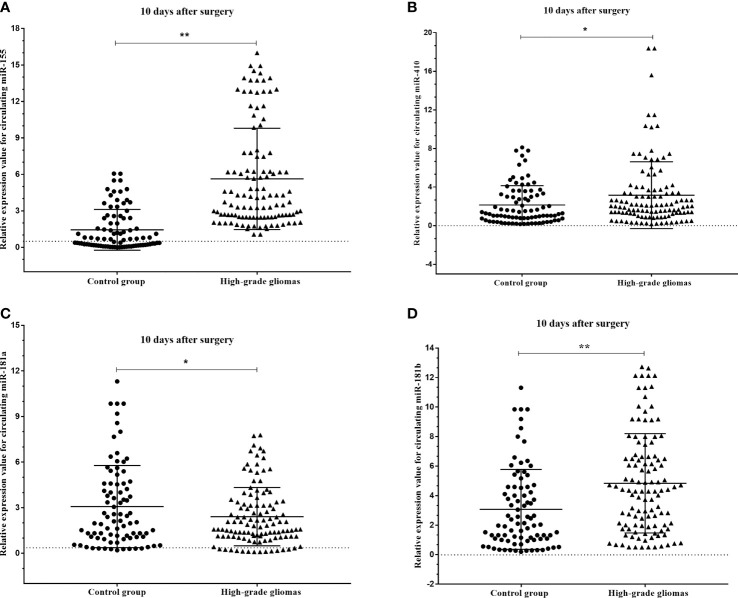
Box plots showing the expression levels of **(A)** miR-155, **(B)** miR-410, **(C)** miR-181a and **(D)** miR-181b before and after 10 days surgery of patients with high-grade glioma with compare control group. A probability of p < 0.05 (*) or p < 0.001 (**) was considered statistically significant.

The results of the circulating miRNA analysis of plasma samples high-grade glioma patients acquired pre- and post-operatively (10 days after surgery) indicated significant upregulation of circulating miR-181a (p=0.045), miR-181b (p<0.001) and miR-410 (p=0.017), and insignificant downregulation of miR-155 (p<0.001) with compare control group (**Figures 5A–D**).

### 3.3 Circulating miRNA Expression in Paired Before vs. After Surgery Plasma Samples From Patients With High-Grade Glioma

**Figure 6:**
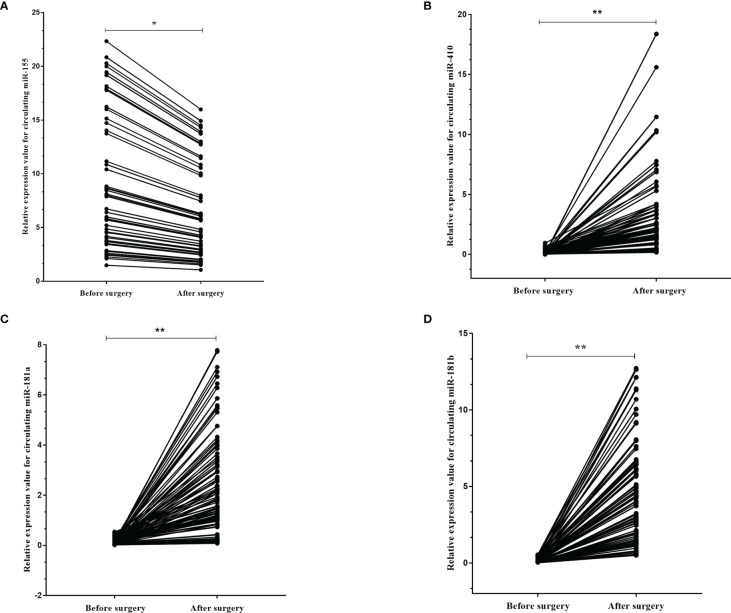
The dynamic change of circulating miR-181a/b, miR-410 and miR-155 in plasma samples of high-grade glioma patients before and after surgery. The expression levels of the 4 circulating miRNAs in the patients before and after surgery **(A–D)**. Each point represents the mean of the triplicate samples. A probability of p < 0.05 (*) or p < 0.001 (**) was considered statistically significant.

We next asked if these circulating miRNAs were different in the plasma of the high-grade glioma patients before and after surgery. We identified that the expression levels of miR-155 (p=0.001) were found to be decreased in the plasma 10 days after surgery compared to that before operation, while miR-181a (p<0.001), miR-181b (p<0.001) and miR-410 (p<0.001) were increased more than ten-fold in the plasma after surgery (**Figures 6A–D**).

### 3.4 Diagnostic Value of Circulating miRNAs in Glioma Patients

**Figure 7:**
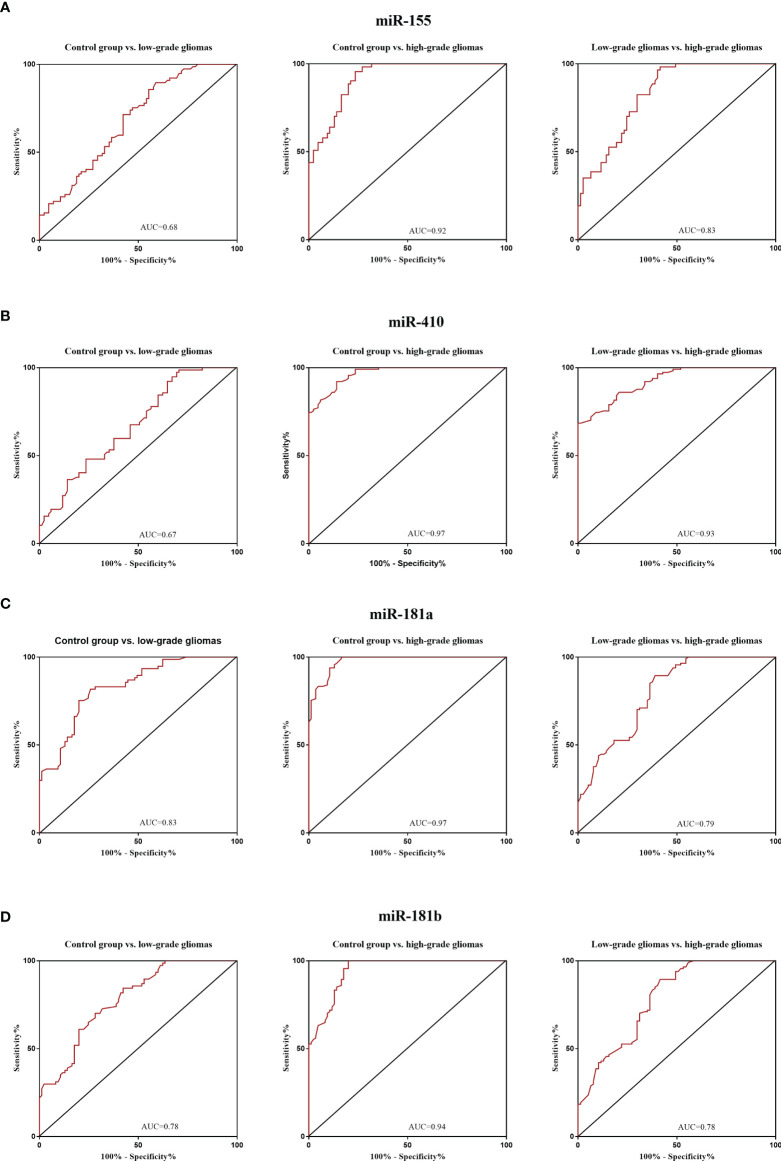
Receiver Operating Characteristic (ROC) curves for circulating miR-181a/b, miR-410 and miR-155 for glioma patients before surgery **(A–D)**. In panel **(A, B)** of the figure the area under the curves (AUCs) for circulating miR-155 and miR-410 were 0.68, 0.92, 0.83, 0.67, 0.97, 0.93 and in panel **(C, D)** for circulating miR-155 and miR-410 were 0.83, 0.97, 0.79, 0.78, 0.94, 0.78 respectively.

**Figure 8:**
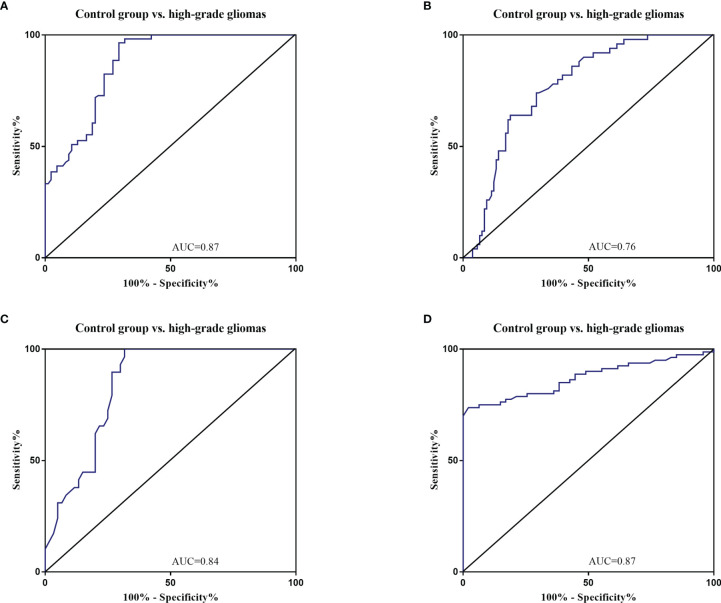
Receiver Operating Characteristic (ROC) curves for circulating miR-181a/b, miR-410 and miR-155 for high-grade glioma patients after surgery **(A–D)**. The area under the curves (AUCs) for circulating miR-155 **(A)**, miR-410 **(B)**, miR-181a **(C)**, and miR-181b **(D)** were 0.87, 0.76, 0.84, and 0.87, respectively, suggesting that these circulating miRNAs can distinguish high-grade glioma patients after surgery from control group.

The diagnostic value in before and after surgery of circulating miR-181a/b, miR-410 and miR-155 in differentiating the high-grade glioma patients group from the low-grade glioma patients and control group was also examined (**Figures 7A–D**, **8A–D**). Our circulating miRNA-based signature could perform well in distinguishing high-grade glioma patients in before and after surgery time from control group as evidenced by a high AUC (diagnostic value AUC≥0.75) (16). These findings suggest that circulating miR-181a/b, miR-410 and miR-155 had high power to distinguish high-grade glioma patients in before and after surgery time from low-grade glioma patients and control group. More detailed information about the ROC curves for diagnostic value of circulating miRNAs are presented in **Tables 3**, **4**.

**Table 3:** Receiver Operating Characteristic (ROC) curves for diagnostic value of circulating miR-181a/b, miR-410 and miR-155 for glioma patients in before surgery time.

miRNAs	Area Under the ROC Curve (95% CI)	Sensitivity (%)	Specificity (%)	p value
Control group vs. low-grade				
miR-155	0.68 (95% CI: 0.6019 to 0.7636)	66.7	76.9	p<0.0001
miR-410	0.67 (95% CI: 0.5898 to 0.7533)	65.7	74.1	p<0.0001
miR-181a	0.83 (95% CI: 0.7671 to 0.8902)	73.34	86.00	p<0.0001
miR-181b	0.78 (95% CI: 0.7122 to 0.8499)	68.21	82.75	p<0.0001
Control group vs. high-grade				
miR-155	0.92 (95% CI: 0.882 to 0.9573)	82.3	84.1	p<0.0001
miR-410	0.97 (95% CI: 0.9467 to 0.9858)	86.8	94.21	p<0.0001
miR-181a	0.97 (95% CI: 0.9602 to 0.9927)	87.5	96.7	p<0.0001
miR-181b	0.94 (95% CI: 0.9154 to 0.975)	93.1	88.7	p<0.0001
Low-grade vs. high-grade				
miR-155	0.83 (95% CI: 0.7688 to 0.89)	73.24	85.95	p<0.0001
miR-410	0.93 (95% CI: 0,8885 to 0,9573)	90.2	82.4	p<0.0001
miR-181a	0.79 (95% CI: 0.7303 to 0.8618)	70.21	83.75	p<0.0001
miR-181b	0.78 (95% CI: 0.719 to 0.8534)	68.51	83.11	p<0.0001

ROC, Receiver Operating Characteristic; CI, Confidence interval.

**Table 4:** Receiver Operating Characteristic (ROC) curves for diagnostic value of circulating miR-181a/b, miR-410 and miR-155 for high-grade glioma patients in after surgery time.

miRNAs	Area Under the ROC Curve (95% CI)	Sensitivity (%)	Specificity (%)	p value
Control group vs. high-grade				
miR-155	0.87(95% CI: 0.8211 to 0.9225)	66.7	76.9	p<0.0001
miR-410	0.76 (95% CI: 0.6956 to 0.8436)	65.7	74.1	p<0.0001
miR-181a	0.84 (95% CI: 0.7617 to 0.9205)	73.34	86.00	p<0.0001
miR-181b	0.87 (95% CI: 0.8087 to 0.9312)	68.21	82.75	p<0.0001

ROC, Receiver Operating Characteristic; CI, Confidence interval.

### 3.5 Expression Levels of Circulating miR-181a/b, miR-410 and miR-155 Predicts a poor Prognosis of High-Grade Glioma Patients

**Figure 9:**
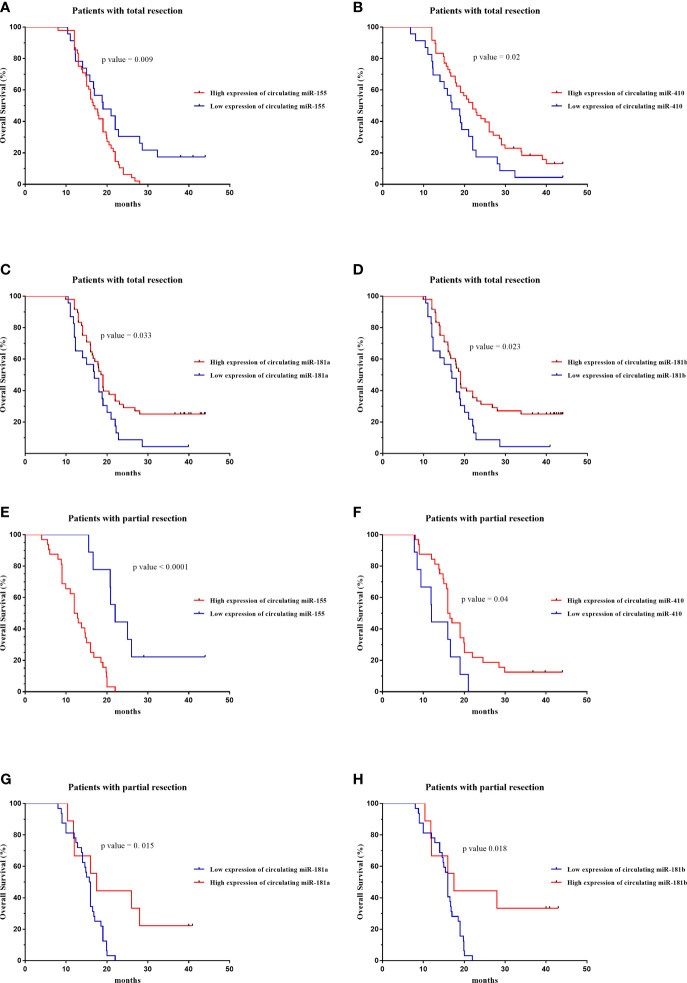
Kaplan–Meier curves of overall survival (OS) for high-grade glioma patients based on the circulating miR-155, miR-410, miR-181a and miR-181b signature in patients with total (n = 71) **(A–D)** or partial tumor resection (n = 43) **(E–H)**.

The prognostic value of circulating miR-181a/b, miR-410 and miR-155-based signature in overall survival (OS) was detectable through the Kaplan–Meier curve of two cohorts (total tumor resection or partial tumor resection) of high-grade glioma patients as shown in (**Figures 9A–H**). The relative expression of these circulating miRNAs in high-grade glioma patients were divided into a higher-expression group and a lower-expression group. Our analysis showed that high-grade glioma patients in the higher-expression group of circulating miR-155 had a poorer OS (**Figure 9A**; p= 0.001) particularly in high-grade glioma patients with partial tumor resection (**Figure 9E**; p=0.05). On the other hand, high-grade glioma patients in the lower-expression group of circulating miR-181a, miR-181b, miR-410 had a poorer OS (**Figures 9B–D**; p=0.001) particularly in high-grade glioma patients with partial tumor resection (**Figures 9F–H**; p=0.05). Based on these findings, we suggest that circulating miR-181a/b, miR-410 and miR-155 expression can be used as an independent factor to predict the survival of high-grade glioma patients.

### 3.6 Interaction Between Potential Gene-Targets and miRNAs

We screened out the possible gene-targets of the aforementioned circulating miRNAs by using common miRNA target predicting datasets: DIANA-microT, mirSVR, PicTar5, RNA22, RNAhybrid, TargetScan, PITA, MirTarget2, TargetMiner, miRanda, and ТmiRWalk2.0. In order to improve the reliability of the predicted target genes, we extracted only the corresponding target regulations that emerged from at least five of the datasets listed above. The selected eight genes, targeted by the miRNAs panel, were MET, phosphatidylinositol 3-kinase (PI3KCA), Wnt family member 5A (WNT5A), programmed cell death 4 (PDCD4), DNA fragmentation factor subunit alpha (DFFA), epidermal growth factor receptor (EGFR), fibroblast growth factor receptor 1/2 (FGFR1/2). Primer sequences for each gene are provided in **Table 2**.

#### 3.6.1 Relationship Between Expression Circulating miRNAs and Expression Gene-Targets of High-Grade Gliomas Patients

**Figure 10:**
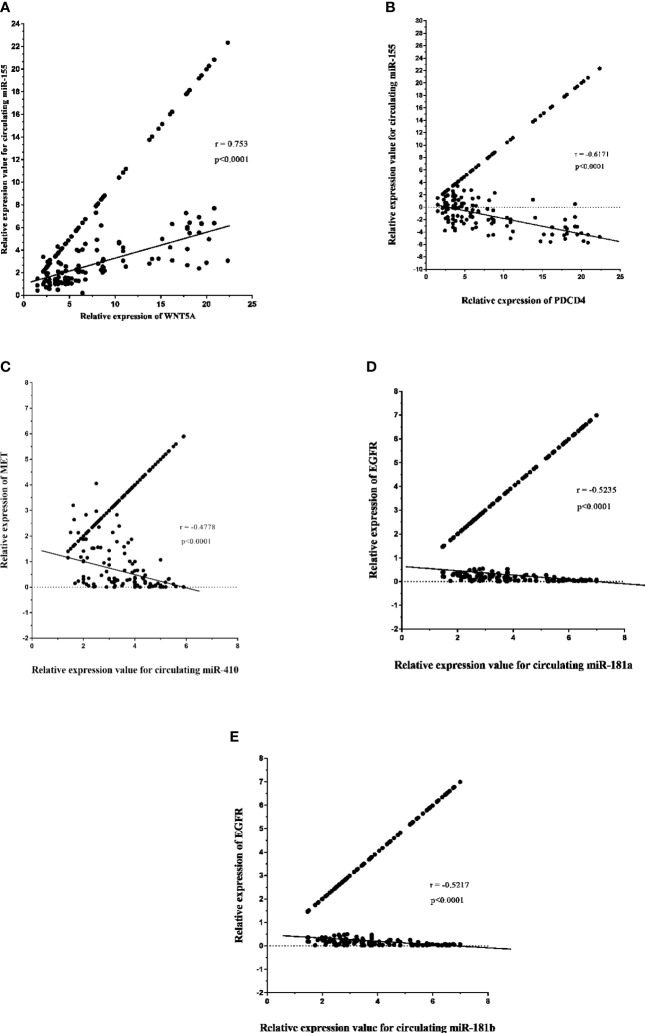
Relative expression of circulating miR-181a/b, miR-410 and miR-155 and its potential gene-targets, programmed cell death 4 (PDCD4), Wnt family member 5A (WNT5A), MET, and epidermal growth factor receptor (EGFR), in plasma samples from high-grade glioma patients **(A–E)**. **(A, B)** Correlation between circulating miR-155 expression and PDCD4 and WNT5A levels in plasma samples (p < 0.0001; Spearman rank correlation test: r = 0.753, r = -0.671). **(C)** Correlation between miR-410 expression and MET levels in in plasma samples (p < 0.0001; Spearman rank correlation test: r = -0.4778). **(D, E)** Correlation between circulating miR-181a/b expression and EGFR levels in plasma samples (p < 0.0001; Spearman rank correlation test: r = -0.5235, r = -0.5217).

The expression level of circulating miR-155 correlated positively with oncogene PDCD4 (p < 0.0001, r = 0.753) in plasma samples of high-grade glioma patients (**Figure 10A**). However, circulating miR-155, miR-410, and miR-181a/b showed a negative correlation with tumor suppressive gene WNT5A (p < 0.0001, r = -0.671; **Figure 10B**) and with oncogenes MET (p < 0.0001, r = -0.4778; **Figure 10C**), EGFR (p < 0.0001, r = -0.5235, r = -0.5217; **Figures 10D, E**). Consequently, the expression of circulating miR-155, miR-410, and miR-181a/b and its putative gene-targets PDCD4, WNT5A, MET, and EGFR had a strong inverse relation in high-grade glioma, thereby suggesting that the potential roles of miR-155, miR-410, and miR-181a/b in oncogenesis of glioma tumors.

## 4 Discussion

Gliomas are the most common and aggressive type of primary brain tumor in adults. It should be noted that for gliomas, the progression of the disease to a higher class of anaplasia is not excluded (e.g. anaplastic astrocytoma and glioblastoma) (3). Despite all the achievements of modern medicine, the prognosis for patients with high-grade gliomas remains unsatisfactory. Current treatment strategies of high-grade gliomas are based on open surgery, radio- and chemotherapy (1, 3). However, none of these treatments, alone or in combination, is considered effective in controlling the disease, resulting in an average life expectancy after diagnosis of about than 12-15 months. Thus, the issues of timely diagnosis and prognosis of the outcomes of this type of tumors do not lose their relevance. The concept of molecular biomarkers has been widely developed in the last decade. The use of biomarkers contributes to the understanding of the pathogenetic mechanisms of gliomas, promotes early detection of tumors, stratification of the risk of recurrence, control and timely correction of the treatment strategy, and, consequently, a more favorable prognosis. Circulating miRNAs are one of the widely studied biomarkers, and although they are not currently used in clinical practice, advances in this field indicate that the effectiveness of circulating miRNAs in the diagnosis and prognosis of high-grade gliomas can be critical and replace specific steps in modern diagnostic practice (15).

In this study, expression levels of circulating miR-155, miR-410, and miR-181a/b in 114 high-grade glioma patients, 77 low-grade glioma patients and 85 healthy volunteers as control group were first screened using qRT-PCR arrays. We found that the expression levels of circulating miR-155 were significantly higher in high-grade glioma patients than in low-grade glioma patients in the before surgery period and control group. Wherein, the expression levels of circulating miR-410 and miR-181a/b were significantly lower in high-grade glioma patients than in low-grade glioma patients in the before surgery period and control group. In addition, using ROC curve analysis, we demonstrated that the circulating miR-155, miR-410, and miR-181a/b had high accuracy in glioma diagnosis, especially in patients with high-grade gliomas in before surgery where AUC ≥0.75, respectively (see **Table 3**) (16). Furthermore, we saw that in the 10 days after surgery the expression levels of circulating miR-155 were insignificantly lower in high-grade glioma patients while and in the same patients group there was a significantly decrease in the expression levels of circulating miR-410, and miR-181a/b than in the control group. The AUC for the diagnosis value of circulating miR-155, miR-410, and miR-181a/b after surgery was 0.87, 0.76, 0.84, and 0.87, respectively (see **Table 4**). In addition, to analyze the association of these circulating miRNAs expression with prognosis, the Kaplan-Meier analysis showed that higher circulating miR-155 the expression levels and lower circulating miR-410 and miR-181a/b the expression levels showed a shorter OS in high-grade glioma patients. Wherein in the patients group with partial tumor resection with higher-expression of circulating miR-155 and lower-expression miR-410 and miR-181a/b had a poorer OS than in the patients with total tumor resection. To the best of our knowledge, the present study was the first comprehensive study on the expression and clinical significance of the expression levels of panel circulating miR-155, miR-410, and miR-181a/b in high-grade glioma patients.

Previous studies *in vitro* and *in vivo* have identified that miR-155, miR-410, and miR-181a/b is important miRNAs in regulating the oncogenesis in glioma, including high-grade gliomas. For instance, Wu et al. demonstrated that miR-155 could effectively accelerate proliferation, migration and invasion of U-87 MG cell line through targeting the PI3K/AKT signaling pathway (8). In other study, using data from The Cancer Genome Atlas (TCGA) dataset for clinical information of 480 glioblastoma samples had demonstrates that miR-155 could serve as prognostic and predictive biomarkers for survival of glioblastoma patients (17). Moreover, miR-155 highly expressed in glioma cells and demonstrated to regulate caspase-3 gene expression, were the target of PNA-based induction of apoptosis in the TMZ-resistant T98G glioma cell line (10). MiR-410, and miR-181a/b has tumor suppressive roles in glioma, and their reduced expression leads to abnormalities in cellular processes, such as an increase in apoptosis, enhanced cell growth, invasion and decreased sensitivity to radio- and chemotherapy through negative suppression of oncogene function (18–22). Wang et al., showed that miR-410 was significantly down-regulated in glioma tissues and in glioma cell lines (U87MG, SF126, LN229, and U251MG). These results indicate that decreased expression of miR-410 correlates with poor prognosis of glioma patients. In additional, the authors indicated that miR-410 exerts tumor-suppressing functions (inhibitory effects on tumor cell proliferation, migration, and invasion) in glioma by directly targeting mRNA 3′-untranslated regions (3′UTR) Ras-related protein 1A (RAP1A) (23). Wang et al. demonstrated that long non-coding RNA (lncRNA) colon cancer-associated transcript-1 (CCAT1) promoted U251 cell line proliferation and colony formation, induced the cell cycle arrest in G0/G1 phase and promoted the tumor cells apoptosis *via* inhibiting miR-410. In other words, these results indicated that miR-410 mediated the tumor-suppressive effects of CCAT1 knockdown on glioblastoma (24). Zhang et al. indicated that upregulation of miR-181b targets B-cell lymphoma 2 (Bcl-2) directly and may function as an important modifier to sensitize U87MG and U251 cells line to TMZ (25). Shi et al. demonstrated that miR-181a and miR-181b are low expression in human gliomas (WHO Grade 1-4) and glioma cell lines, U87, TJ905, and U251 (13). Moreover, miR-181a and miR-181b has great biological effect on tumor cells growth, proliferation, invasion and apoptosis, and may function as tumor suppressors in high-grade gliomas.

Given the roles of miR-155, miR-410, and miR-181a/b in the glioma oncogenesis, we used the online tools DIANA-microT, mirSVR, PicTar5, RNA22, RNAhybrid, TargetScan, PITA, MirTarget2, TargetMiner, miRanda, and ТmiRWalk2.0 to predict the possible genes candidate as targets for these miRNAs. The selected eight genes, targeted by the miRNAs panel, were MET, PI3KCA, WNT5A, PDCD4, DFFA, EGFR, and FGFR1/2. As result, the current study showed that circulating miR-155 correlated positively with PDCD4 in plasma of high-grade glioma patients. In the same time, miR-155, miR-410, and miR-181a/b correlated negatively with WNT5A, MET, and EGFR in plasma of high-grade glioma patients. Latest reports have shown that these miRNAs by regulation PDCD4, WNT5A, MET, and EGFR also serves certain biological function in the progression of various human tumors including glioma. For instance,

These results further suggest that miR-155, miR-410, and miR-181a/b may play a significant role in the development and progression of glioma by regulation PDCD4, WNT5A, MET, and EGFR. Future studies *in vitro* and *in vivo* may address whether targeting miR-155, miR-410, and miR-181a/b and these potential gene-targets may provide a novel therapeutic strategy to suppress the progression of high-grade gliomas.

In summary, ours is the first study to systematically interrogate the clinical significance of panel circulating miR-155, miR-410, and miR-181a/b in glioma, and we provide comprehensive evidence that miR-155, miR-410, miR-181a/b may act as an oncomiR and tumor suppressive miRNAs, as well as a non-invasive diagnostic and prognostic biomarkers in gliomas, particularly high-grade gliomas.

## 5 Conclusions

We verified that increased the expression levels of circulating miR-155 and reduced of circulating miR-181a/b and miR-410 are a prospective candidate non-invasive biomarker for high-grade glioma diagnosis and prognosis. Moreover, miR-155, miR-181a/b and miR-410 may participate in the molecular mechanism of high-grade glioma by interaction PDCD4, WNT5A, MET, and EGFR. Confirming the potential role of miR-155, miR-181a/b and miR-410 in the oncogenesis of glioma and confirming it as non-invasive biomarkers in diagnosis and prognosis of high-grade glioma requires more clinical trials and *in vitro* and *in vivo* studies.

## Data Availability Statement

The raw data supporting the conclusions of this article will be made available by the authors, without undue reservation.

## Ethics Statement

The studies involving human participants were reviewed and approved by The approval was provided by the ethics committee of Federal Center of Neurosurgery (Tyumen, Russia). Written informed consent to participate in this study was provided by the participants’ legal guardian/next of kin. Written informed consent was obtained from the individual(s), and minor(s)’ legal guardian/next of kin, for the publication of any potentially identifiable images or data included in this article.

## Author Contributions

Conceptualization, Writing - original draft and Project administration: JW, AA-Z and OB. Writing - review and editing, Investigation and Resources: RS and IG. Formal analysis and Methodology: SM and GS. Data curation: AS. Validation and Visualization: RT. Funding acquisition: JW. Supervision: AS. All authors have read and agreed to the published version of the manuscript.

## Conflict of Interest

The authors declare that the research was conducted in the absence of any commercial or financial relationships that could be construed as a potential conflict of interest.

## Publisher’s Note

All claims expressed in this article are solely those of the authors and do not necessarily represent those of their affiliated organizations, or those of the publisher, the editors and the reviewers. Any product that may be evaluated in this article, or claim that may be made by its manufacturer, is not guaranteed or endorsed by the publisher.
